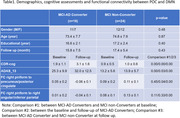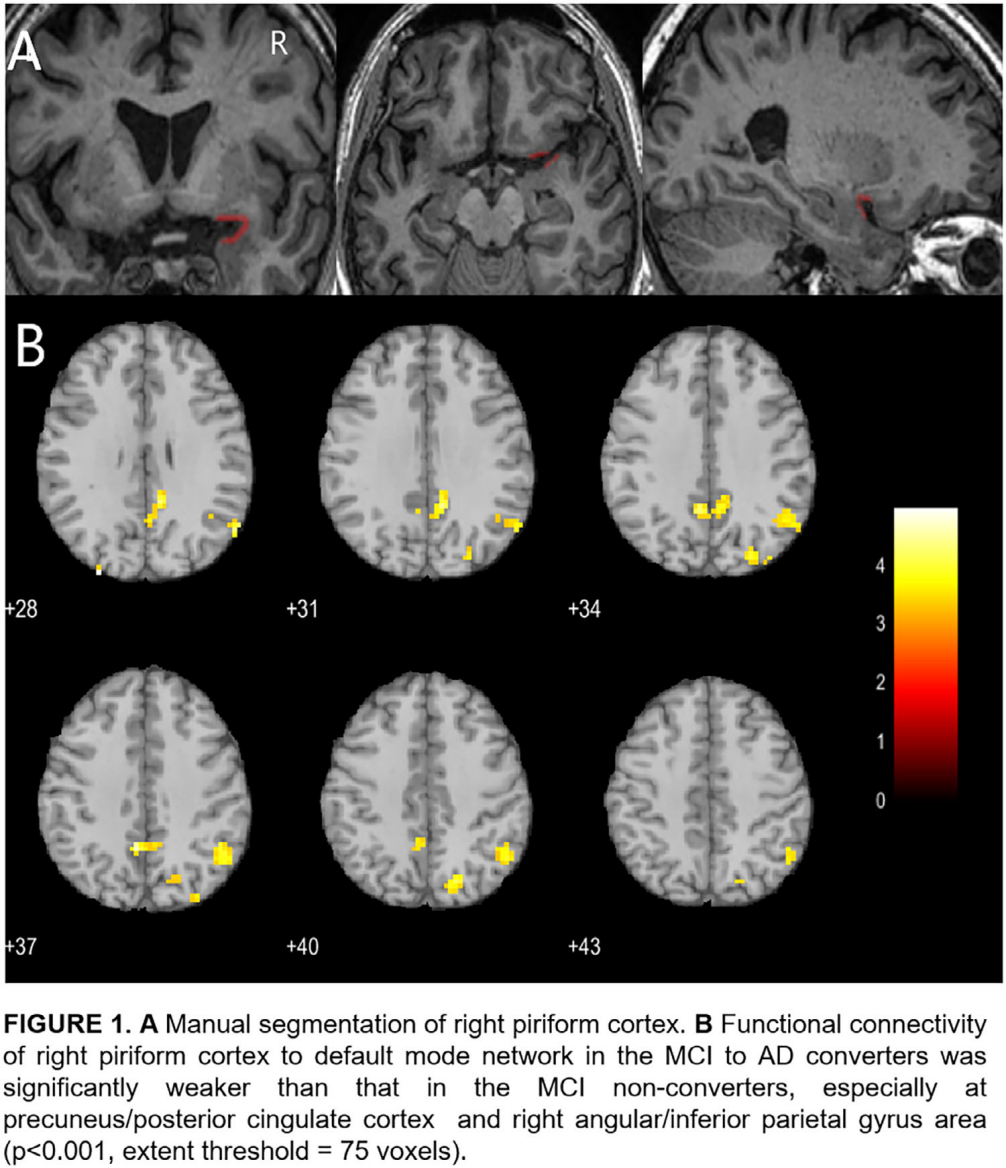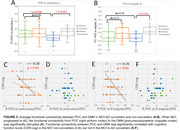# An Imaging Marker for MCI to AD Conversion: Functional Dysconnectivity between Primary Olfactory Cortex to Default Mode Network

**DOI:** 10.1002/alz.088384

**Published:** 2025-01-09

**Authors:** Ran Pang, Jianli Wang, Prasanna Karunanayaka, Sangam Kanekar, Paul Eslinger, Jens Will, Deepak Kalra, Biyar Ahmed, Rommy Elyan, Qing Yang

**Affiliations:** ^1^ Pennsylvania State University College of Medicine, Hershey, PA USA; ^2^ Department of Radiology, Pennsylvania State University College of Medicine, Hershey, PA USA; ^3^ Departments of Neurosurgery and Radiology, Pennsylvania State University College of Medicine, Hershey, PA USA

## Abstract

**Background:**

Resting‐state functional MRI (rs‐fMRI) has been widely used for assessing disease progression in AD. It becomes, however, notoriously variable at disease’s prodromal stage when the cognitive functions are fluctuating while declining at the same time during this transitional period, which reduces its sensitivity in AD detection. Olfactory deficit has been shown to be an early sign of AD when the cognition decline is indistinguishable from normal aging. In this study, we investigated our hypothesis that functional connectivity (FC) between olfactory network and default mode network (DMN) will be disrupted in early AD.

**Method:**

Longitudinal rs‐fMRI data of 42 clinically diagnosed MCI were collected at 1st‐time point from ADNI database (Table 1). The piriform cortex was manually segmented as the seed of POC (Figure 1A). The FC between POC and DMN was evaluated and analyzed within and between MCI‐AD converters and non‐converters.

**Result:**

Compared to MCI non‐converters, AD patients’ FC between POC and DMN was significantly weaker (p<0.05, Table 1 and Figures 1‐2), while there was no significant difference in regards of POC‐DMN FC between MCI‐AD converters and non‐converters at the baseline (p>0.05). When the disease was progressed to AD, the FC between right piriform cortex and precuneus/PCC was significantly reduced (p=0.04). The FC of POC to DMN in MCI non‐converters was significantly correlated with cognitive score CDR‐cog, but not in MCI‐AD converters (Figure 2).

**Conclusion:**

The FC between POC and DMN was disrupted in early AD. The observed disruption established a mechanistic link between olfactory deficits and cognition decline in AD. The functional dysconnectivity between POC and DMN could be an objective imaging marker for MCI conversion to AD.